# RRegrs: an R package for computer-aided model selection with multiple regression models

**DOI:** 10.1186/s13321-015-0094-2

**Published:** 2015-09-15

**Authors:** Georgia Tsiliki, Cristian R. Munteanu, Jose A. Seoane, Carlos Fernandez-Lozano, Haralambos Sarimveis, Egon L. Willighagen

**Affiliations:** School of Chemical Engineering, National Technical University of Athens, 9 Heroon Polytechneiou Street, Zografou Campus, 15780 Athens, Greece; Computer Science Faculty, University of A Coruna, Campus Elviña, s/n, 15071 A Coruña, Spain; Department of Bioinformatics-BiGCaT, NUTRIM, Maastricht University, P.O. Box 616, UNS50 Box 19, 6200 MD Maastricht, The Netherlands; Stanford Cancer Institute, Stanford University, C.J.Huang Building, 780 Welch Road, Palo Alto, CA 94304 USA

**Keywords:** Multiple regression, QSAR, R package, Caret-based tool

## Abstract

**Background:**

Predictive regression models can be created with many different modelling approaches. Choices need to be made for data set splitting, cross-validation methods, specific regression parameters and best model criteria, as they all affect the accuracy and efficiency of the produced predictive models, and therefore, raising model reproducibility and comparison issues. Cheminformatics and bioinformatics are extensively using predictive modelling and exhibit a need for standardization of these methodologies in order to assist model selection and speed up the process of predictive model development. A tool accessible to all users, irrespectively of their statistical knowledge, would be valuable if it tests several simple and complex regression models and validation schemes, produce unified reports, and offer the option to be integrated into more extensive studies. Additionally, such methodology should be implemented as a free programming package, in order to be continuously adapted and redistributed by others.

**Results:**

We propose an integrated framework for creating multiple regression models, called RRegrs. The tool offers the option of ten simple and complex regression methods combined with repeated 10-fold and leave-one-out cross-validation. Methods include Multiple Linear regression, Generalized Linear Model with Stepwise Feature Selection, Partial Least Squares regression, Lasso regression, and Support Vector Machines Recursive Feature Elimination. The new framework is an automated fully validated procedure which produces standardized reports to quickly oversee the impact of choices in modelling algorithms and assess the model and cross-validation results. The methodology was implemented as an open source R package, available at https://www.github.com/enanomapper/RRegrs, by reusing and extending on the caret package.

**Conclusion:**

The universality of the new methodology is demonstrated using five standard data sets from different scientific fields. Its efficiency in cheminformatics and QSAR modelling is shown with three use cases: proteomics data for surface-modified gold nanoparticles, nano-metal oxides descriptor data, and molecular descriptors for acute aquatic toxicity data. The results show that for all data sets RRegrs reports models with equal or better performance for both training and test sets than those reported in the original publications. Its good performance as well as its adaptability in terms of parameter optimization could make RRegrs a popular framework to assist the initial exploration of predictive models, and with that, the design of more comprehensive in silico screening applications.

**Graphical abstract:**
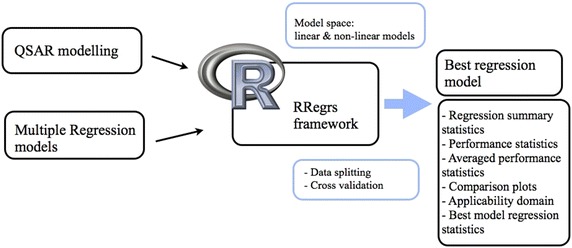
RRegrs is a computer-aided model selection framework for R multiple regression models; this is a fully validated procedure with application to QSAR modelling

## Background

Many open-source statistical, data-mining, and machine learning software projects give access to a wide range of data processing and modelling algorithms often providing graphical user interfaces. Among them are Weka (Waikato Environment for Knowledge Analysis) [1], RapidMiner [2], Keel (Knowledge Extraction based on Evolutionary Learning) [3], Orange [4], Scikit-learn [5], C1C2 [6] and KNIME (Konstanz Information Miner) [7], effectively, providing full predictive modelling frameworks.

Additionally, web-based platforms such as OpenTox [8] and Online Chemical Modelling Environment (OCHEM) [9] focus on the development of quantitative structure-activity relationship (QSAR) models, i.e. regression or classification models that are used for the in silica assessment of physicochemical properties and biological activities of chemical compounds such as toxicity, biological potency and side effects [10–12]. Such platforms typically consist of two major subsystems: the database of experimental measurements and the modelling framework. They allow users to create their own QSAR models, predict results for new chemicals, and share them. OpenTox in particular is a platform-independent collection of components which communicate through web services, so that the user can combine data, models and validation results from multiple sources in a dependable and time-effective way. Several other tools offer virtual evaluation of chemical properties and toxicity using implemented QSAR models; for instance, Vega [13], EPI Suite [14], Toxicity Estimation Software Tool (TEST) [15], QSAR4u [16], BuildQSAR [17], OECD QSAR Toolbox [18], AZOrange [19] or Bioclipse-R [20]. However, these tools are limited to supported data sets, QSAR models or specific regressions methods.

On the other hand, the R statistical language environment [21] offers many solutions for regression modelling and also some packages providing simultaneous access to multiple methods. For example, the glmulti package conducts automated model selection and model-averaging based on the Akaike Information Criterion (AIC) or the Bayesian Information Criterion (BIC) [22], the kernlab package applies Kernel-based machine learning methods for classification, regression, clustering, novelty detection, quantile regression and dimensionality reduction, and the e1071 package includes functions for latent class analysis, short time Fourier transform, fuzzy clustering, and support vector machines. Other R packages are specializing on particular set of algorithms, for instance the R package tree focuses on producing classification and regression trees.

Of particular interest is the R package for predictive modelling called caret (Classification and Regression Training) which gathers and simplifies numerous R algorithms for the development of a wide variety of predictive models by calling and integrating more than 25 other packages [23]. Unique features of caret include data splitting, pre-processing, characterizing performance and variable importance, and parallel processing tools.

Although this package is providing useful methods for the syntactical unification of regression and classification prediction modelling approaches, the various models have different inputs and the outcomes different formats, typically depending on their parameters. This makes it hard to run all the available methods for multiple data sets, compare all the outputs, and produce a standardized results summary.

Furthermore, the complexity of the workflows complicates the reproduction of the same regression results and it may affect decisions on issues, such as how to split the original data set, how often to split the data set, which cross-validation method is to be use, which data filtering to apply before regression (correlated features removal, “not available” (NA) values elimination, etc.), which data scaling is applied (normalization, standardization, etc.), which regression methods to test, which regression parameters and seeds to use, how to summarize and compare the results for several regression models, and which criteria to use in order to choose the best regression model.

To address these limitations, the current manuscript describes a standardized framework that automates the development of a reliable and well-validated QSAR model, or set of models. The so-called RRegrs tool (R Regressions) is based on the R caret package and is focusing on regression modelling. RRegrs workflow offers a fully validated procedure capturing any variability or inconsistency in the data. A single RRegrs function call is needed to run the entire workflow and obtain the produced validated QSAR model(s) in a reproducible format in contrast to the standard, inefficient and time-consuming QSAR modelling workflow, where the modeller tries many different algorithms and even needs to further search the parameter space of each algorithm. This is a considerable advantage for users with perhaps limited statistical knowledge or limited R experience. Also reproducibility is a comparative advantage since often the same procedure needs to be applied for different data sets. RRegrs implements an easy way to explore the models’ search space of linear and non-linear models with special parameters specifications and cross validation schemes. Furthermore, model outputs are easily accessible and readable, organized by methods, centralized and averaged by multiple reproducible data set splits. Summary files are also produced helping the user to easily access all methodologies results, which can then be prioritized based on various statistics. The current implementation of the RRegrs package contains ten different regression algorithms and supports parallel processing, if prompted. RRegrs function calls can be integrated into complex desktop and web tools for QSAR. RRegrs package is available as an open repository at https://www.github.com/enanomapper/RRegrs. The current release is available from ZENODO with the doi:10.5281/zenodo.21946.

## Results and discussion

RRegrs is an R package for computer-aided model selection, designed and implemented as a collection of regression tools available from the caret package. RRegrs uses the R package testthat for testing [24]. It does not apply full unit testing, but several RRegrs parameter combinations are tested, which are run during the build process. RRegrs can be used to find the best regression model for any numerical data set using some or all of ten linear and non-linear regression models: Multiple Linear regression (LM), Generalized Linear Model with Stepwise Feature Selection (GLM) [25], Partial Least Squares Regression (PLS) [26], Lasso regression (LASSO) [27], Elastic Net regression (ENET) [28], Support vector machine using radial functions (SVRM) [29], Neural Networks regression (NN) [30], Random Forest (RF) [31], Random Forest Recursive Feature Elimination (RF-RFE) [32] and Support Vector Machines Recursive Feature Elimination (SVM-RFE) [33]. Using the above methods, we explore the model space and compare outputs to decide on the optimal model, given the data. We are setting the regression method parameters with grid functions which have been carefully chosen to optimize different models, and particularly this is done for PLS, SVRM, SVM-RFE, NN, RF, RF-RFE, ENET. Specifically for SVRM, SVM-RFE the user could also specify custom parameters.

The main scope of the presented tool is to be able to run a large number of regression methods from the caret package using only one function call, to use standardized cross-validation (CV) scheme for all the methods, to obtain standardized outputs, to generate result summary tables and comparison plots for the regression methods and to store for each method detailed statistics and fitting plots. RRegrs integrates results of individual models and decides on the best model given the data set and the user specified parameters, unlike caret. Therefore, RRegrs permits users, irrespective of their programming or statistics experience, to predict any type of numerical output using multiple regression methods. In addition, advanced users could integrate RRegrs in other applications or software packages. For example, in cheminformatics RRegrs can be used to generate predictive models using molecular descriptors calculated with the rcdk package, using functions offered by the Chemistry Development Kit [34].

**Fig. 1:**
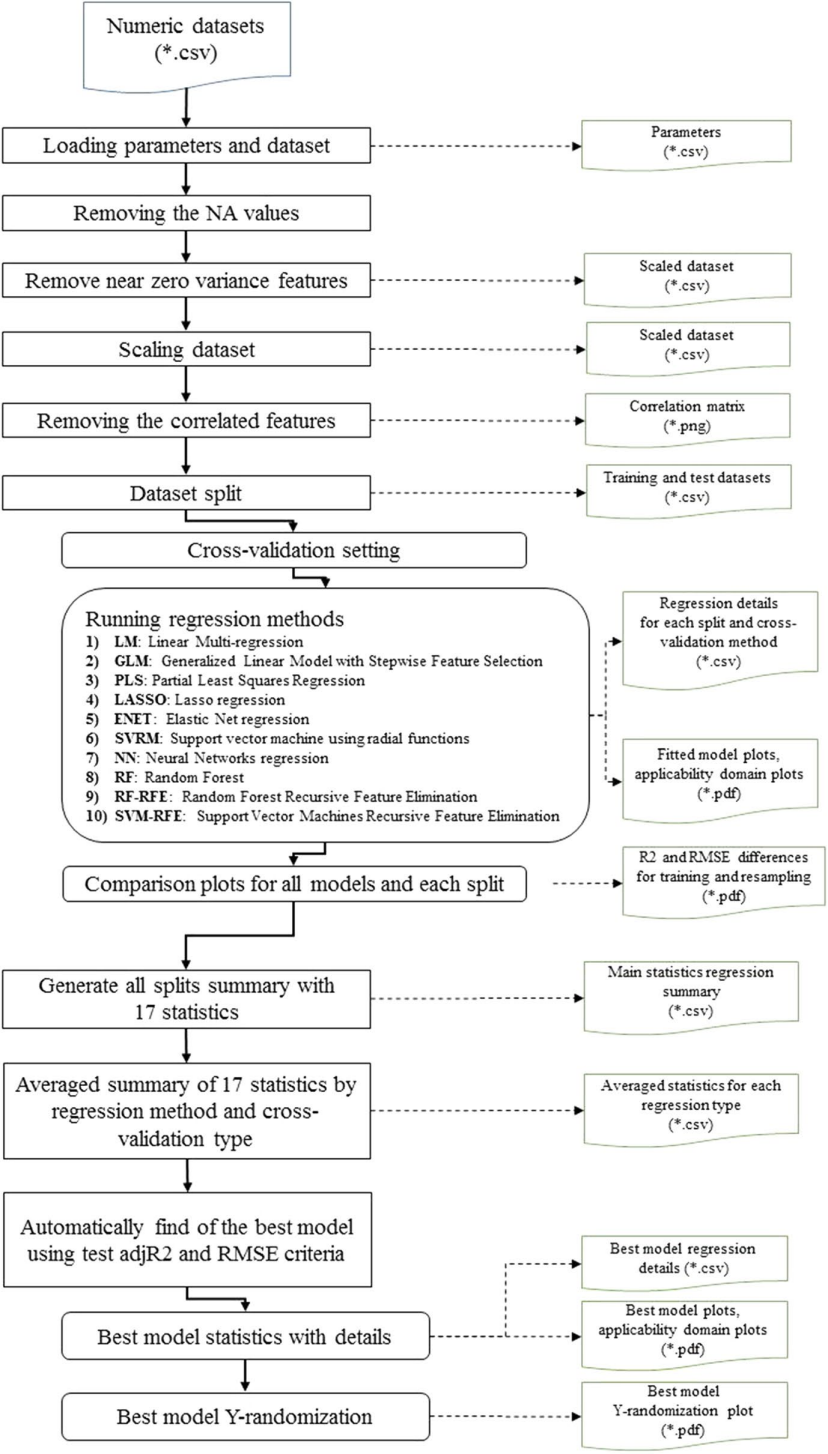
RRegrs methodology flowchart. Outline of the steps performed by the RRegrs function

RRegrs function wraps up all the above mentioned procedures within just one call. The following steps are included (see Fig. 1): load parameters and data set, remove the NA values, remove near zero variance features, scale the data set, remove correlated features, split data set into training and test sets, run the selected regression methods using the selected cross-validation method(s), summarize the statistics for all methods and splittings, average for each method and cross-validation type for all splittings, apply the best model of each method and split on the test sets, apply Y-randomization on the best model, and assess the Applicability Domain. For each model, a CV scheme is introduced with two options: 10-fold repeated CV and Leave-One-Out (LOO) CV. The more time-consuming regression methods (RF, SVM-RFE, RF-RFE) are using only repeated cross-validation (other validation methods could be very slow for these complex functions), whereas for computationally demanding methods RRegrs offers parallel processing for a defined number of CPU cores. Detailed output files for all regression methods are produced, plots for individual model statistics, as well as summary statistics and comparison plots between methods, resulting in a significant number of CSV, PDF, PNG outputs files. Additionally, several summary files are created. A CSV output file is created with all the basic statistics (17 values) for each method, data splitting and CV type. Based on the above, averaged statistics are calculated for each regression method and across all data splits, which are the values needed to decide on the final best model performance. The best model is further validated with Y-randomization runs (100 by default).

For each regression method, caret package utilities are employed. For example, RRegrs uses the trainControl and train functions to set the training conditions (10 repetitions; RMSE used as metrics to choose the model) and train the model, respectively. For each method, RRegrs is generating the same list of 17 statistics values: regression name, split number, cross-validation type, number of model features, names of model features, training adjusted R-squared (adj.R^2^), training root mean squared error (RMSE_CV/LOO_), training ${\text{R}}_{{{\text{CV}}/{\text{LOO}}}}^{2}$, training standardized RMSE, test adjusted R^2^, test RMSE (RMSE_test_), test R^2^ (${\text{R}}_{\text{test}}^{2}$), test correlation, and corresponding values for both sets. If the user requests detailed output (the default flag is set to True), several files are generated such as a CSV file with statistics about each regression model listing the following information: regression method, splitting number, cross-validation type, training set summary, test set summary, fitting summary, list of predictors, training/test predictors, a full list of statistics as defined above, feature importance, residuals of the fitted model, assessment of applicability domain/leverage analysis such as mean of hat values, hat values with warnings, leverage threshold, list of points with leverage greater than threshold, Cook’s distances, Cook’s distance cutoff, points influence. Particularly, for each data splitting and CV method, the following plots are produced: observed versus predicted values for training/test sets, feature importance, fitted versus residuals for the fitted model, leverage statistics for fitted model, Cook’s distance for fitted model, and six standard fitting plots including Cook’s distance cutoff.

Moreover, RRegrs offers an exhaustive validation framework by introducing multiple random data splittings. For each algorithm and data split, the model is produced based on training and validation sets. We are reporting both CV and external validation statistics, however, the test set is used to select the final best model, i.e. the best performing amongst the optimal models produced by different algorithms (both linear and non-linear). Decision is made on the averaged statistics across data splits to remove any bias towards the structure of the test set. The best regression model is selected based on the following criterion: from the best averaged ${\text{R}}_{\text{test}}^{2}$ (+/−0.05), the model with minimum RMSE_test_ is the final one. Alternatively, the test adjusted R^2^ can be used to select the best model. For the best model, an additional CSV file is generated providing detailed statistics as well as PDF plots for important statistics.

As mentioned above, parallel processing is employed during training steps by enabling caret’s parallel design, and it is activated by either using caret’s TrainControl “allowParallel” option, or in the case of RFE methods also within the model selection (iterating with a parallel foreach through the cross validation model selection for each feature size) using RFEControl “allowParallel” option. Libraries doMC (Linux/Mac) and doSNOW (Windows) provide foreach parallel adaptor.

The uses of RRegrs reported here are aimed at finding QSAR models for cheminformatics and nanotoxicology for the eNanoMapper European FP7 project. RRegrs was first tested with five standard data sets from UC Irvine Machine Learning Repository [35], followed by a demonstration the efficiency of RRegrs in cheminformatics and bioinformatics areas, using three publicly available data sets, as presented in the following sections.

### RRegrs models for standard data sets

To benchmark RRegrs we first used five standard regression data sets from the UC Irvine machine learning repository [35]: the housing [36], computer hardware, wine quality [37], automobile [38], and Parkinsons telemonitoring [39] data sets. Based on these data sets we demonstrate the RRegrs methodology capabilities in different scientific fields. The number of cases and features of these data sets are described in the Methods section.

The housing data set is the most used standard data set for complex regression methods: combination of regression estimators as genetic algorithm-based selective neural network ensemble [40], distributed multivariate regression using wavelet-based collective data mining [41], application of the Bayesian evidence framework to support vector regression (SVR) [42], Principal Components approach that combines regression estimates [43], regression on feature projections (RFP) method [44], subset-based least squares subspace regression in reproducing Kernel Hilbert space (RKHS) [45], Smola and Scholkopf’s sequential minimal optimization (SMO) algorithm for SVM regression [46], etc.

**Fig. 2:**
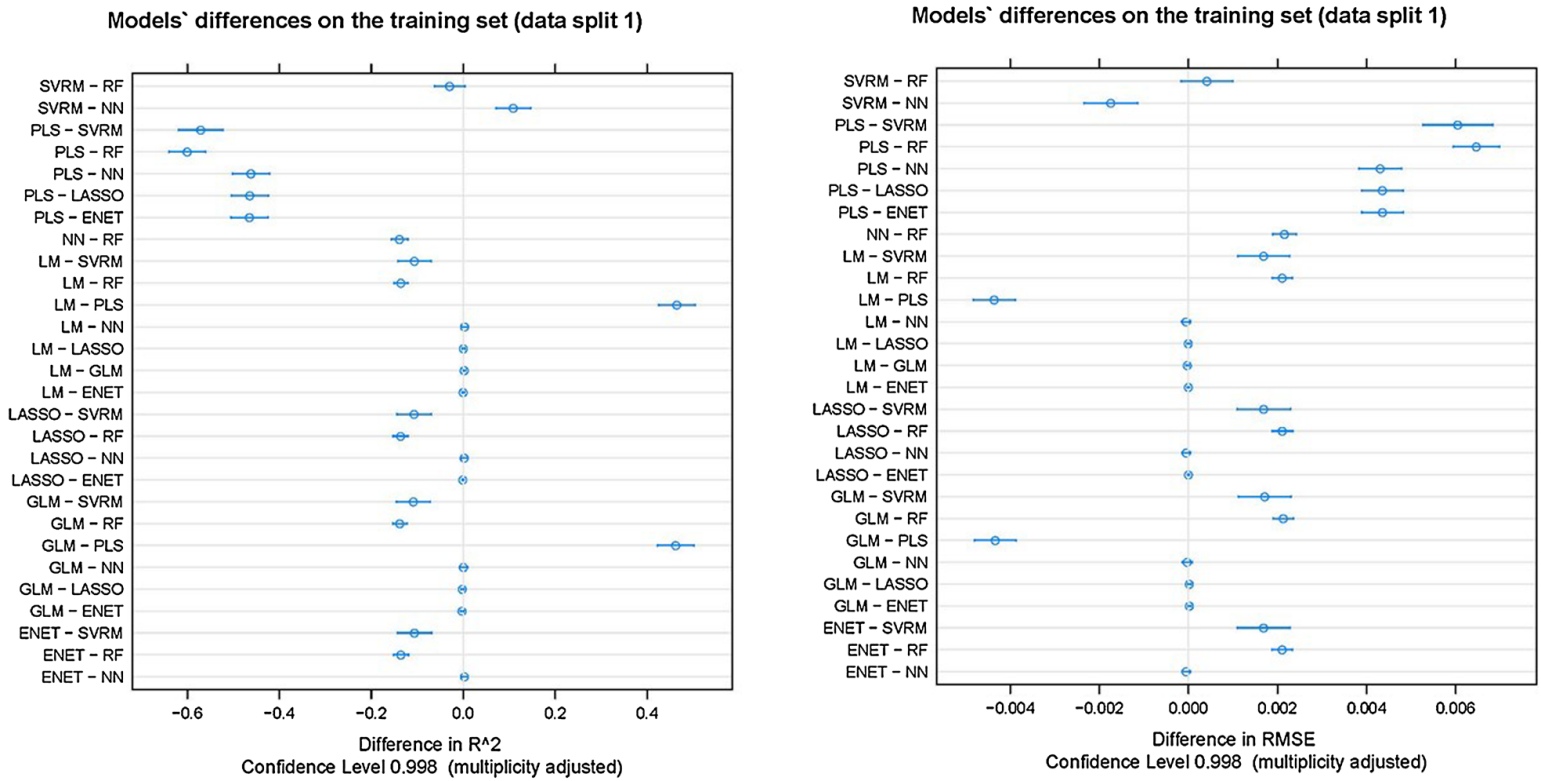
Models’ differences on the Housing training set (data split 1). We show the average performance value (*dot*) with two-sided confidence limits as computed by Student *t* test with Bonferroni multiplicity correction. Results are shown for RMSE and R^2^ statistics and all pairwise model comparisons

**Fig. 3:**
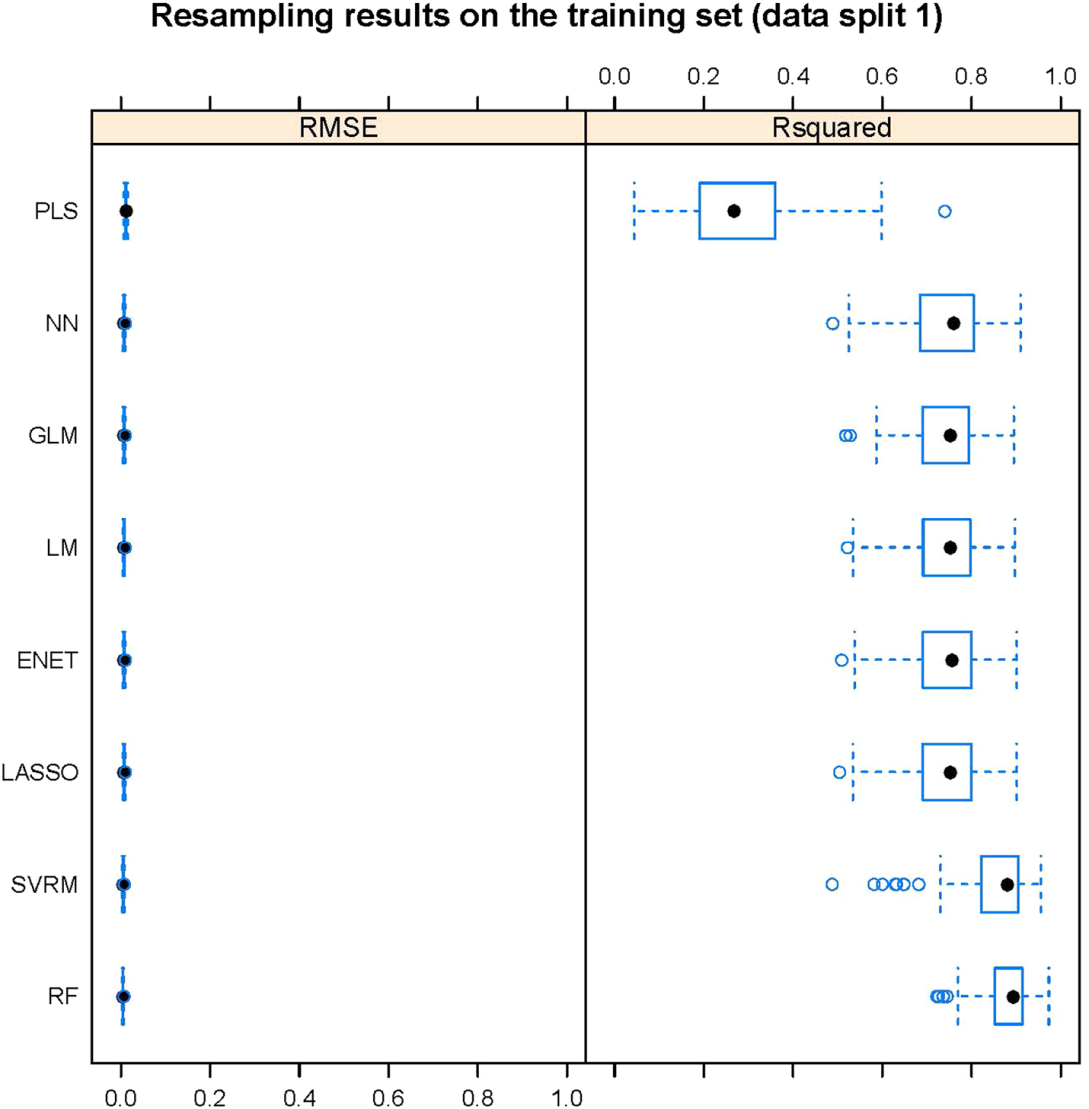
Models’ comparison of resampling results for Housing training set (data split 1). Univariate visualization of the resampling distributions of RMSE and R^2^ statistics, for the various RRegrs models

**Fig. 4:**
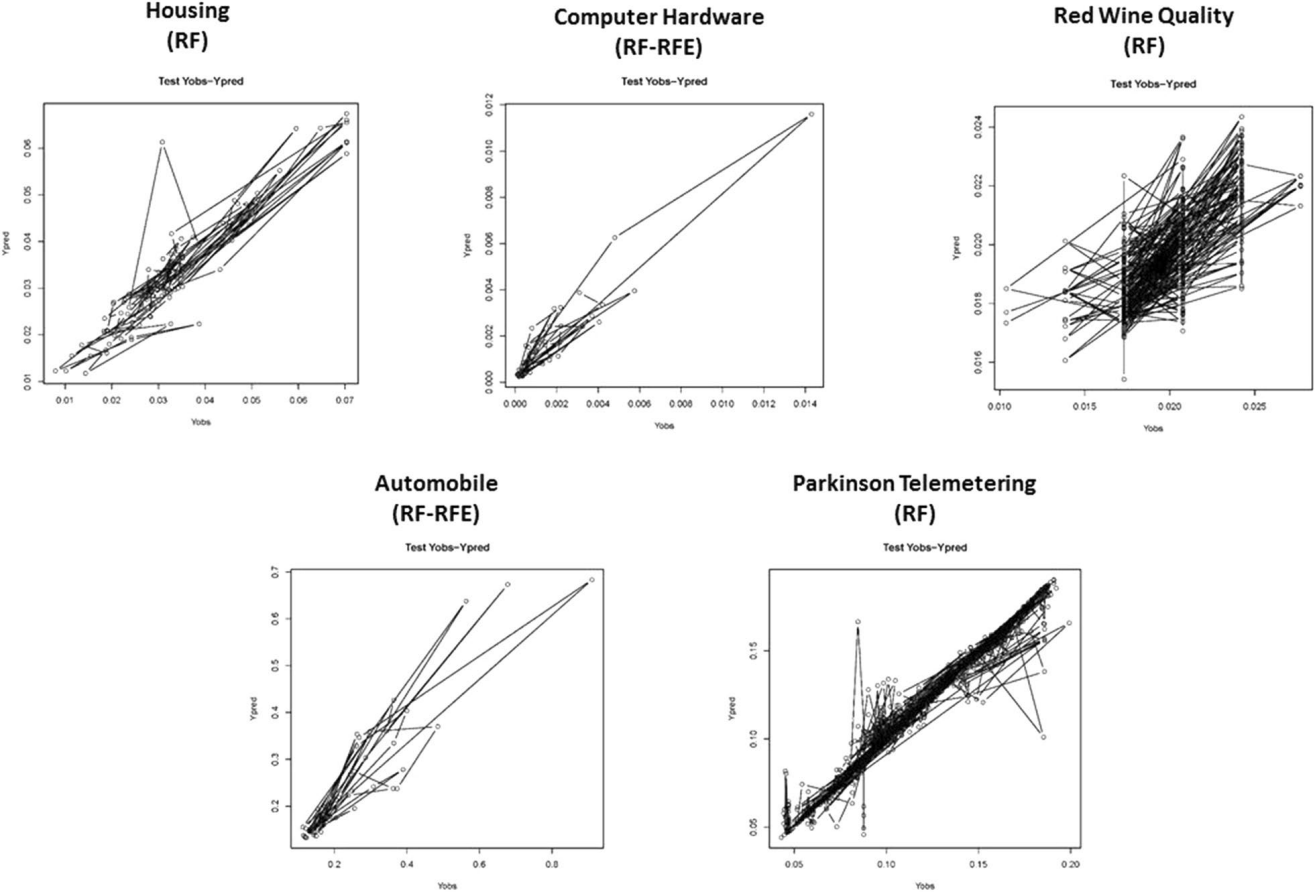
Test Yobs—Ypred for the five standard data sets best models (10-fold cross-validation). Plots for the observed versus the predicted values and the best model found for each of the five data sets

**Fig. 5:**
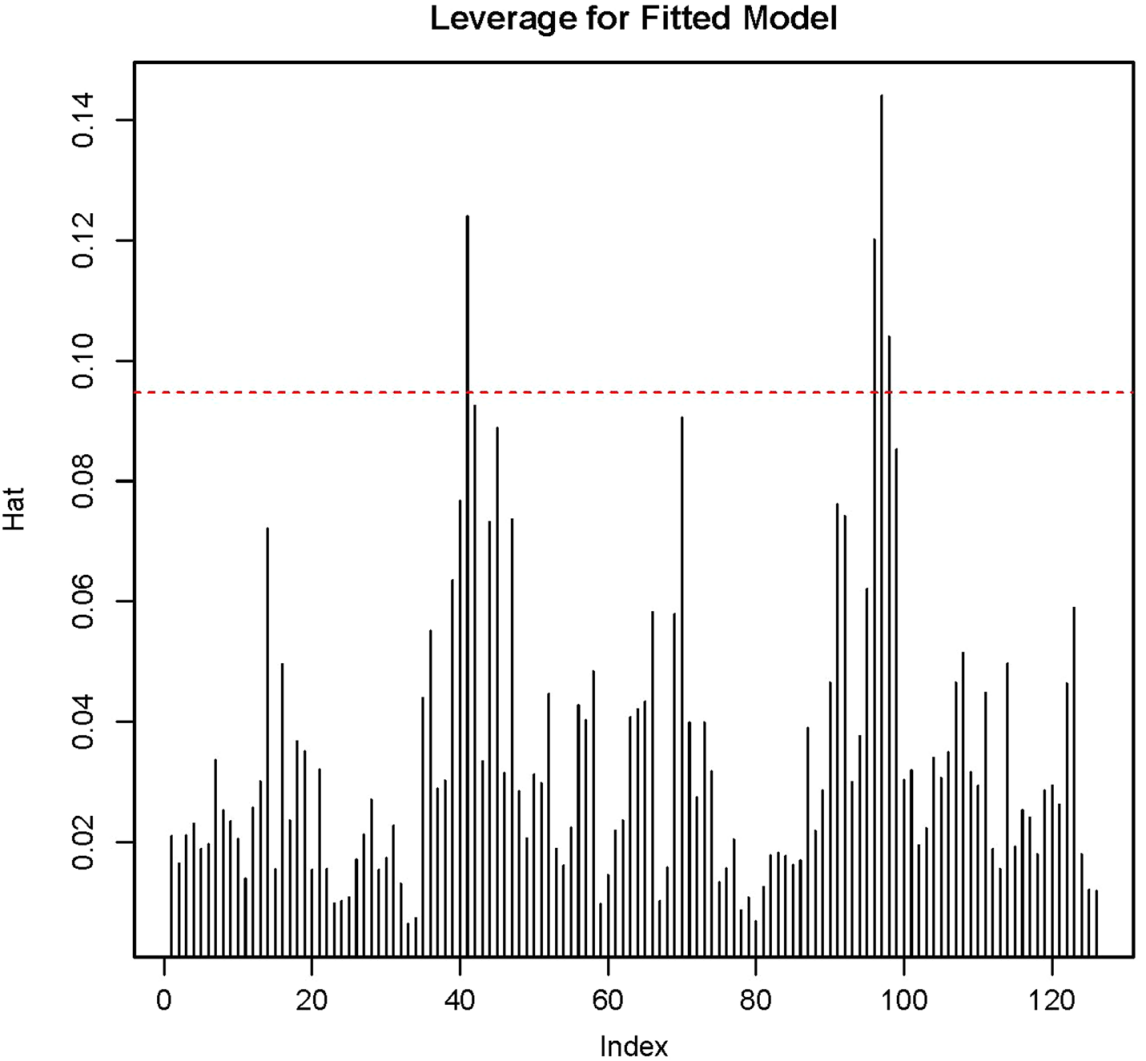
Leverage for Housing best fitted model. Histogram showing the Hat values for the RF fitted model. The* red dashed line* indicates the leverage threshold value ($\frac{{3{\text{m}}}}{\text{n}}$, where m are the number of model parameters and n the number of observations)

Tables 1 and 2 present two statistic values for these standard data sets: ${\text{R}}_{\text{test}}^{2}$ and RMSE_test_ values, averaged by 10 different data set splits, using 10-fold repeated CV and 10 Y-randomization. The results show that advanced methods such as RF-RFE and RF give the highest R^2^ values. In the case of the Housing data set, PLS provides a very low ${\text{R}}_{\text{test}}^{2}$ of 0.266 compared with the RF-RFE/RF that shows 0.875/0.874 (${\text{R}}_{\text{test}}^{2}$ for LM is 0.707). Because of its slightly lower RMSE_test_ value compared to RF-RFE (less than 0.001), RRegrs suggests RF as the best model. Figure 2 shows the differences for ${\text{R}}_{\text{test}}^{2}$ and RMSE_CV_ on the training set (data split 1) and Fig. 3 presents the comparison for resampling on the training set (data split 1). In order to observe the quality of the regression models, Fig. 4 presents the observed versus predicted values in the test set for the best models for five data sets (10-fold repeated CV). The applicability domain section of RRegrs plotted the leverage for the Housing best fitted model (RF) as in Fig. 5.

**Table 1:** Test averaged R^2^ values for five standard data sets

RRegrs method	Housing	Computer hardware	Red wine quality	Automobile	Parkinson telemonitoring
LM	0.707	0.822	0.355	0.824	0.154
GLM	0.709	0.825	0.353	0.824	0.153
PLS	0.266	0.740	0.066	0.757	0.091
LASSO	0.704	0.828	0.354	0.831	0.154
ENET	0.705	0.826	0.354	0.830	0.154
SVRM	0.845	0.765	0.396	0.853	0.637
NN	0.688	0.824	0.352	0.829	0.142
RF	0.874	0.907	0.500	0.915	0.972
RF-RFE	0.875	0.903	0.501	0.914	0.900
SVM-RFE	0.717	0.742	0.383	0.714	0.479

**Table 2:** Test averaged RMSE values for five standard data sets

RRegrs method	Housing	Computer hardware	Red wine quality	Automobile	Parkinson telemonitoring
LM	0.007	0.001	0.002	0.076	0.034
GLM	0.007	0.001	0.002	0.076	0.034
PLS	0.011	0.001	0.003	0.094	0.035
LASSO	0.007	0.001	0.002	0.074	0.034
ENET	0.007	0.001	0.002	0.075	0.034
SVRM	0.005	0.001	0.002	0.067	0.022
NN	0.007	0.001	0.002	0.075	0.034
RF	0.005	0.001	0.002	0.052	0.006
RF-RFE	0.005	0.001	0.002	0.052	0.013
SVM-RFE	0.008	0.002	0.002	0.113	0.027

The best model for the Computer Hardware data set was obtained with the RF method (${\text{R}}_{\text{test}}^{2}$ of 0.907) and the worst one using PLS (${\text{R}}_{\text{test}}^{2}$ of 0.740). The LM method has ${\text{R}}_{\text{test}}^{2}$ of 0.822. Models for the Red Wine data set do not produce good values for ${\text{R}}_{\text{test}}^{2}$ (>0.501) due to the non-continuous values of the output variable. When RRegrs is applied to the Automobile data set, ${\text{R}}_{\text{test}}^{2}$ values vary from about 0.915 for RF/RF-RFE to 0.714 for SVM-RFE (${\text{R}}_{\text{test}}^{2}$ for LM is 0.824).

If the RRegrs call uses all available CPU cores for the complex methods, one dataset split, one Y-randomizations, and all the regression methods, the following execution times (in seconds) are obtained for the Boston standard dataset (see Table 3). The computer was an Windows 8.1 64bit with i7-4790 CPU (3.60 GHz, 4 cores, 8 logical cores), 16G RAM. The total execution time was 5.43 min (325.64 s).

**Table 3:** RRegrs execution time (in seconds) for one split of Boston House dataset

Method	Repeated CV	LOOCV
LM	11.97	1.78
GLM	2.14	5.48
PLS	0.99	1.40
Lasso	1.32	–
ENET	12.70	45.30
SVM radial	4.62	13.77
NN	12.53	49.97
RF	88.89	–
RF-RFE	3.89	–
SVM-RFE	46.36	–

### Use case 1: RRegrs application on protein corona data

Recent studies have shown that the presence of serum proteins in vitro cell culture systems form a protein adsorption layer (a.k.a. the ‘protein corona’) on the surface of nanoparticles (NPs). This corona is reported to affect the nanoparticle-cell interactions as well as change the cell response [47, 48], and defines the NP’s ‘biological identity’ [49]. It thus encodes information about the interface formed between the NP core and the cell surface within a physiological environment.

This section presents results for proteomics data recently published that characterizes the serum protein corona ‘fingerprint’ formed around a library of 105 distinct surface-modified gold NPs [49]. The authors used LC–MS/MS to identify 129 serum proteins which were considered suitable for relative quantification. The relative abundances for each of these proteins on the corona of a nanoparticle formulation defines the serum protein ‘fingerprint’ for that formulation. The authors presented a multivariate regression model that uses the protein corona fingerprint to predict cell association for the gold NPs and found a model predicted cell association with a ${\text{R}}_{\text{LOO}}^{2}$ of 0.81. Specifically, they applied a PLS regression model along with an internal iterative filtering procedure using the Variable Importance to the Projection (VIP) score and jackknife resampling.

Here we present results on the initial set of 129 × 84 proteins to gold NPs data (21 neutral NPs were excluded from analysis as in Walkey et al. [49]), and also on a set of 76 × 84 proteins to gold NPs data. These 76 proteins are selected in [49] with VIP ≥ 0.6 threshold. RRegrs was applied with 10 random splits of the data (75 % train and 25 % test) along with 10 Y-randomization runs for the best model. Table 4 shows the best model selected by RRegrs, its number of features, the adj.R^2^ and the R^2^ and RMSE values for the train and test sets, averaged over 10 random splits of the data. Table 5 shows the best model found in all data splits, i.e. we compare all methodologies and data splits to find the best R_test_^2^. Data are normalized and filtered using the RRegrs near zero variance and correlation filters, for that reason the 129 proteins are filtered to be 99 and the 76 proteins data set are reduced to 60 features.

**Table 4:** RRegrs averaged statistics reported for the three use cases, under the 10-fold repeated CVscheme

Use case	Best model	Features no.	adj.R^2^	${\text{R}}_{\text{CV}}^{ 2}$	${\text{R}}^{ 2}_{\text{test}}$	RMSE_CV_	RMSE_test_
UC1: protein corona							
129 proteins	SVRM	99	1.02	0.687	0.631	0.558	0.612
76 proteins	SVRM	60	0.582	0.777	0.728	0.477	0.538
UC2: metal oxides	ENET	8.8	1	0.933	0.746	0.639	0.639
UC3: toxicity data	SVRM	8	0.7	0.556	0.537	0.68	0.67

Averaged values are reported across the ten different data splits

**Table 5:** RRegrs best model statistics reported for the three use cases. Both LOO and CV values are considered

Use case	Best model	Data split	Features no.	Validation type	adj.R^2^	${\text{R}}_{\text{CV/LOO}}^{ 2}$	${\text{R}}_{\text{test}}^{ 2}$	RMSE_CV/LOO_	RMSE_test_
UC1: protein corona									
129 proteins	SVRM	5	99	LOO, CV	1.03	0.644/0.61	0.844	0.618/0.643	0.357
76 proteins	SVRM	5	60	LOO, CV	0.407	0.767/0.741	0.89	0.525/0.527	0.296
UC2: metal oxides	ENET	8	8	LOO	0.808	0.7	0.998	0.588	0.246
UC3: toxicity data	SVRM	2	8	LOO	0.685	0.506	0.657	0.705	0.609

**Fig. 6:**
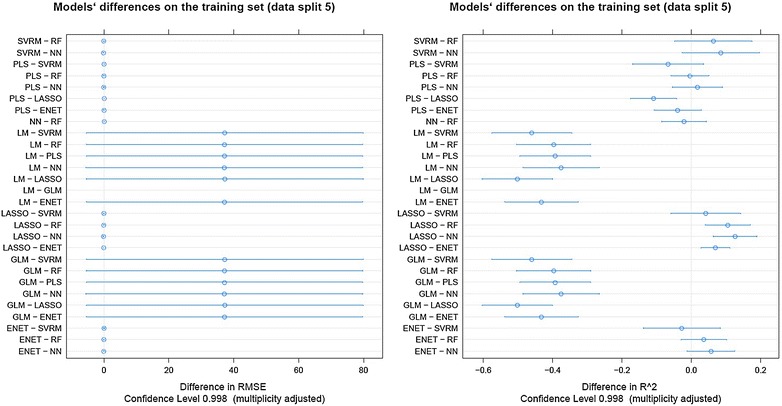
Models’ differences for protein corona training set (data split 5). Differences for R^2^ and RMSE values among the applied models are presented. The average performance with two-sided confidence limits are plotted as derived by the Student t-test with Bonferroni multiplicity correction. We can observe that LM and GLM models are not fitting the data very well (large RMSE values)

For the data set with 129 proteins, the best model is an SVRM model with ${\text{R}}_{\text{test}}^{2}$ = 0.631. CV results on the training set can be seen in Fig. 6, where we can observe that the LM and GLM models are not suitable for the protein corona data, whereas the remaining methodologies perform similarly. It can be observed in Table 5 that the highest value reported was ${\text{R}}_{\text{test}}^{2}$ = 0.844 for individual split nine of the data set.

**Fig. 7:**
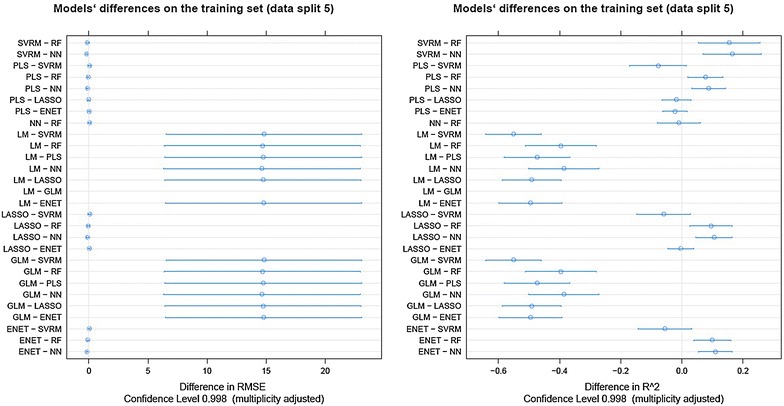
Models’ differences for protein corona optimal training set (data split 5). Differences for R^2^ and RMSE values among the applied models are presented for the trimmed corona data set (76 proteins). The average performance with two-sided confidence limits are plotted as derived by the Student t-test with Bonferroni multiplicity correction

When we study the set with 76 proteins, we find that the best model is an SVRM model with averaged ${\text{R}}_{\text{test}}^{2}$ = 0.728, whereas the best individual split value is ${\text{R}}_{\text{test}}^{2}$ = 0.89. CV results on the training set are shown in Fig. 7. The corresponding RRegrs results for the PLS model are ${\text{R}}_{\text{test}}^{2}$ = 0.7 (averaged over 10 data splits), whereas the highest values are reported for individual split five ${\text{R}}_{\text{test}}^{2}$ = 0.885 (for repeated CV) and ${\text{R}}_{\text{test}}^{2}$ = 0.873 (for LOO). Although the last number cannot be directly compared to ${\text{R}}_{\text{LOO}}^{2}$ = 0.81 reported by the authors, it gives an indication of how our PLS implementation performs for the specific data set.

### Use case 2: RRegrs application on metal oxides data set

The authors of [50] combined experimental and theoretical measurements to develop a nano-QSAR model that describes the toxicity of eighteen nano-metal oxides (MeOx) to the human keratinocyte (HaCaT) cell line, which is a common in vitro model for keratinocyte response during toxic dermal exposure. The study was aimed at exposing and explaining the differences in modes of toxic action of metal oxide nanoparticles between the eukaryotic system and the prokaryotic system (*E. coli*).

They calculated 32 parameters that quantitatively describe the variability of the nanoparticles’ structure, called nano-descriptors, which included quantum-mechanical descriptors derived from quantum-chemical calculations, and image descriptors derived from transmission electron microscopy (TEM) images. Some of the descriptors included are: particle size and size distribution, agglomeration state, particle shape, crystal structure, chemical composition, surface area, surface chemistry, surface charge, electronic properties (reactivity, conductivity, interaction energies, etc.), and porosity. Additionally, the LC_50_ was calculated from experimental data for all MeOx NPs. This is the concentration that caused a 50 % reduction of the cells after 24 h exposure, whereas the −log(LC_50_) values were used in modelling, as the dependent variable t be predicted.

The authors applied a Genetic Algorithm (GA) to independently select the most efficient combination of the molecular descriptors which were then analyzed using multiple linear regression. They found that two descriptors were sufficient to predict NPs toxicity with high statistical significance, namely $\Delta {\text{H}}_{\text{c}}^{\text{f}}$ descriptor, which is the enthalpy of formation of metal oxide nanocluster representing a fragment of the surface and, χ^c^ descriptor, which is the Mulliken’s electronegativity of the cluster. The reported values are: R^2^ = 0.93 (RMSE = 0.12), ${\text{R}}_{\text{LOO}}^{2}$ = 0.86 (RMSE_LOO_ = 0.16), ${\text{R}}_{\text{test}}^{2}$ = 0.83 (RMSE_test_ = 0.13). Note that R^2,^ here and in other cases, refers to the coefficient of determination for fitting the model to the training data.

**Fig. 8:**
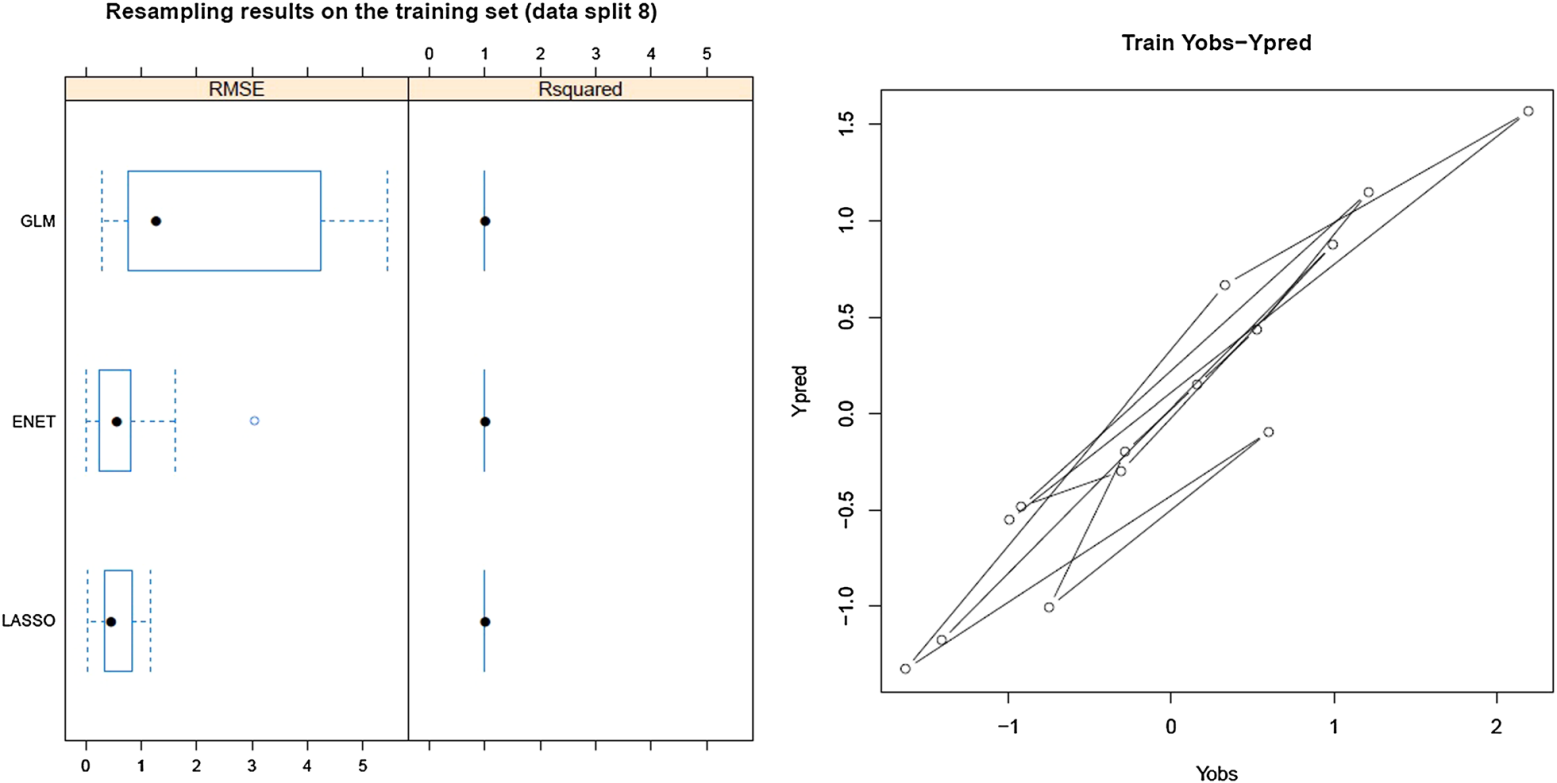
Results on MeOx data set (data split 8). For the three selected methodologies we show the resampling distributions for the RMSE and R^2^ statistics. On the right hand-side, we show the observed versus the predicted values for the ENET model in the training set

Ten random splits of the data were performed (75 % train and 25 % test) along with ten Y-randomization runs for the best model. Tables 4 and 5 show RRegrs results for the initial set of 32 parameters to the eighteen MeOx’s, averaged or non-averaged values across the ten data splits, respectively. Because of the restricted number of samples and descriptors RRegrs was applied without filtering options, whereas data were normalized, as in [50]. The best model was selected between those that perform feature selection, i.e. GLM, LASSO, SVM-RFE, RF-RFE, ENET. As can be seen from the tables, the best performance model and the best averaged model is ENET in this case, keeping on average 8.8 variables from the data including the two important variables (∆H^c^, χ^c^) selected in the original publication. The ENET averaged statistics for 10 splits of the data are ${\text{R}}_{\text{test}}^{2}$ = 0.746, ${\text{R}}_{\text{CV}}^{2}$ = 0.933, which are very similar to the values reported by the authors. The best individual split value is equal to ${\text{R}}_{\text{test}}^{2}$ = 0.998 for ENET model with eight variables including the final two suggested by the authors (LOO at the eighth split of the data). The boxplots in Fig. 8 show the resampling values of RMSE_CV_ and ${\text{R}}_{\text{CV}}^{2}$ values in the training set for split eight, where only methodologies with the same resampling scheme are included in the graph. Figure 8 also includes the fit of the selected model ENET for the same data split.

### Use case 3: RRegrs application on aquatic toxicity data set

The authors of [51] developed a QSAR model based on 546 organic molecules, to predict acute aquatic toxicity towards Daphnia magna, which is the organism preferred for short-term aquatic toxicity testing according to REACH [52]. Ad hoc-designed workflows were used for data curation and filtering, as well as for the extraction of LC_50_ data, which in this case is defined to be the concentration that causes death in 50 % of test Daphnia magna over a test duration of 48 h. For modelling purposes the −log(LC_50_) values were considered as the dependent variable to be predicted. Other experimental data on aquatic toxicity were retrieved from three databases and available scientific publications, as well as one-dimensional (1-D) and2-D molecular descriptors implemented with DRAGON software [51], resulting in a total of 2, 187 molecular descriptors.

A modified k-Nearest Neighbour (kNN) strategy coupled with GA algorithms was used to select the relevant molecular descriptors. The final data set comprised of 546 organic molecules and a set of 201 descriptors. The GA-kNN strategy was implemented with a threshold on the average Mahalanobis distance from the k nearest neighbours, so that only molecules satisfying the threshold criterion were predicted. Particularly, predictions for molecules with an average distance greater than 1.26 from their three neighbours, were considered to be outside of the applicability domain. The training molecules exceeding the threshold did not contribute to the model’s statistics, but were not removed from the data set. The final model showed good performance when the average distance threshold was applied, namely ${\text{R}}_{\text{CV}}^{2}$ = ${\text{R}}_{\text{test}}^{2}$ = 0.78 (5-fold CV), R^2^ = 0.72. The model selected eight molecular descriptors that encoded information about lipophilicity, the formation of H-bonds, polar surface area, polarisability, nucleophilicity and electrophilicity. When no distance threshold is applied, the corresponding values are: R^2^ = 0.60, ${\text{R}}_{\text{CV}}^{2}$ = 0.61, ${\text{R}}_{\text{test}}^{2}$ = 0.43.

**Fig. 9:**
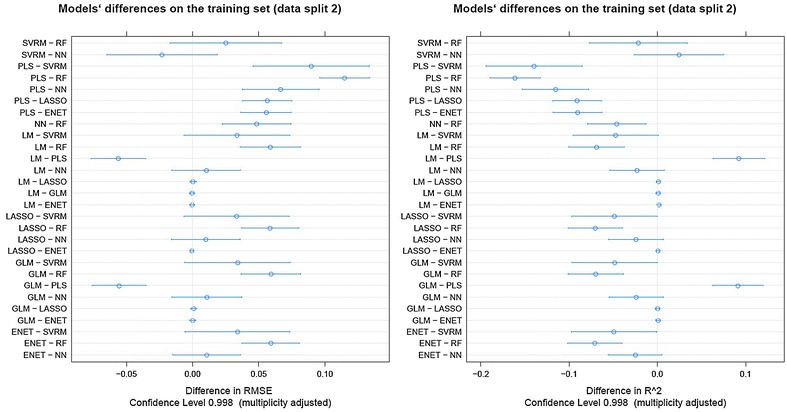
Models’ differences for toxicity training set (data split 2). Differences for R^2^ and RMSE values among the applied models are presented. The average performance with two-sided confidence limits are plotted as derived by the Student t-test with Bonferroni multiplicity correction. We can observe small differences for LM, LASSO, ENET models, whereas the performance of the best model, SVRM, appears to be close to that of RF and NN models

Tables 4 and 5 show the results for the final set of eight descriptors for 546 organic molecules: the averaged values across data splits and the best model statistics for all data splits are presented. RRegrs is applied using normalization and filtering options. Ten random splits of the data were performed (75 % train and 25 % test) along with ten Y-randomization runs for the best model. As can be seen from the tables the best performance model and the best averaged model is SVRM in this case, keeping all 8 descriptors in the data. The SVRM averaged statistics for ten splits of the data are ${\text{R}}_{\text{CV}}^{2}$ = 0.556 (10-fold CV), ${\text{R}}_{\text{test}}^{2}$ = 0.537, which are close to the ones reported by the authors when the distance threshold is not applied. The adjusted R^2^ = 0.7, exceeding the 0.6 value reported without the distance threshold application, and still approaching the 0.78 value when the distance threshold was applied. The best individual split value is reported to be ${\text{R}}_{\text{test}}^{2}$ = 0.657 for SVRM model with eight variables (LOO at the second split of the data). Figure 9 shows the differences between the various models in terms of ${\text{R}}_{\text{CV}}^{2}$ and RMSE_CV_ values in the training set of the second data split, i.e. the train data where the highest ${\text{R}}_{\text{test}}^{2}$ was observed. We can observe that the PLS model differs from all others, having the worst performance, whilst LM, GLM, LASSO, and ENET models have very similar performances.

## Conclusions

This paper introduces RRegrs as a new computer-aided model selection framework using a single R function call. The aim of RRegrs is to automatically obtain the best regression model given the data set, and the set of all ten regression models available, after an extensive search of the model space. A fully validated procedure is suggested where data are split in training and test sets, ten times by default, capturing any variability or inconsistency in the data. The best model is then found across different data splits and cross-validation schemes, based on the averaged data splits statistics. RRegrs produces easily accessible summary files that provide an overview of model details and allows methodology comparisons using the same statistics, enabling QSAR model selection. These direct comparisons are built on top of the caret package, and in that respect provide a useful flexibility for all users. However, use of this package does not require advanced knowledge of R, while, on the other hand, experienced R users can easily modify and extend the package with additional algorithms of choice. The single function call makes it easy to integrate into larger QSAR and in silico molecular screening studies. The new tool was tested with five standard data sets from several domains and three use cases originating from cheminformatics and nanotoxicity, showing good performance in all cases. RRegrs is open source and available from https://www.github.com/enanomapper/RRegrs (doi:10.5281/zenodo.21946).

## Methods

### Regression methods in RRegrs

The RRegrs tool is using ten different linear and non-linear regression models briefly described in this section, to explore the model space. The most basic model in this package is the LM model [33]. Variable selection could improve the result of prediction in regression models. For that reason we have included a generalized linear model, denoted by GLM, which selects variables that minimize the AIC score. LASSO and ENET are also penalizing the number of variables via an embedded minimization process [27, 28].

Apart from the standard regression methodologies included in RRegrs, other methods that focus on specific characteristics of the data are included. PLS uses linear projections of input and output sets, which is a useful strategy when many of the inputs are correlated. PLS coefficient optimization algorithm improves previous regression coefficient algorithms because the search path is directed to high variance and high correlations paths [26]. The SVMR algorithm attempts to find the hyperplane that separates the positive and negative samples, practically allowing a non-linear solution to a regression problem by transforming the data to a hyperdimensional feature space using the kernel functions [53, 54]. SVMR in RRegrs uses the radial function or Gaussian function. RRegrs package also allows the use of a support vector regression model where the w^2^ (the square of SVM hyperplane weight vector) measures the importance of each feature [29].

RRegrs includes two additional learning algorithms, namely NN and ensemble RF. NN is usually defined as a network of a large number of connected neurons (simple processors), which produce good results with imprecise and complicated data [30]. RF is a bagging method constructing decision trees based on the random subspace method [31].

Finally, we have included two of the best performing methodologies with extra feature selection characteristics. Particularly, because the SVM and RF methods can be time-consuming, we have considered their implementation with random feature elimination (RFE), a feature selection method also introduced in caret where less important features are sequentially removed from the model until optimal performance is reached [32]. The two methods are here labeled as RF-RFE and SVM-RFE. Further details for the functions’ main parameters are available in the online tutorial of the RRegrs package.

### Model optimization

Two CV schemes are employed within RRegrs, namely 10-fold repeated CV and LOO CV. In the case of repeated CV, we run ten repeats of 10-fold CV for all models except SVM-RFE (3-folds, one repeat) and RF-RFE (5-folds, one repeat), which are particularly time-consuming methods. The procedure followed by caret and also introduced in RRegrs tool, randomly splits the data in K distinct blocks of roughly equal size (K = 10, 3, 5 depending on the method). Each block of data is left out sequentially, and a model is fit to the remaining of the data; this model is used to predict the held out block. The process is repeated where for each repetition a random proportion of the data are used to train the model (default value is 0.75) while the remainder is used for testing the models. Average performance across the number of repeats are reported: ${\text{R}}_{\text{test}}^{2}$, RMSE_test,_, ${\text{R}}_{\text{CV}}^{2}$, ${\text{R}}_{\text{LOO}}^{2}$, RM SE_C V_, RM SE_LOO_. The best model is selected based on the averaged R_test_^2^ value; if multiple models only differ by ≤0.005 from the best ${\text{R}}_{\text{test}}^{2}$value, the model with the lowest RMSE_test_ statistic is selected.

In order to further validate RRegrs test results, Y-randomization is applied to the best model found. For the last data split and the best model found, RRegrs performs Y-randomization for the 10-fold repeated CV scheme, and compares ${\text{R}}_{\text{test}}^{2}$ values to the best model corresponding value.

### RRegrs tested data sets

RRegrs has been used to find the best regression models for eight data sets: three from nano- and cheminformatics (use cases), and five standard data sets from different fields. The standard data sets have been downloaded from UC Irvine machine learning repository [35]: housing [36], computer hardware, wine quality [37], automobile [38] and Parkinsons telemonitoring [39] data sets. The non-numeric columns have been eliminated, whereas the first column is the dependent variable (output of the model). The number of initial features/cases are the following: 13/506 for Housing data set, 6/209 for Computer Hardware data set, 11/1, 599 for Red Wine Quality data set, 14/195 for Automobile data set, and 19/5, 875 for Parkinson telemonitoring data set. The use case data sets were derived from their original publications; the initial number of features and cases are the following: the protein corona data set [49] has 129 features and 84 cases, the metal oxide data set [50] has 32 features and 18 cases and the toxicity data set [51] contains 8 features, and 546 cases.

### RRegrs call in R

The main function of RRegrs (also called RRegrs) permits the call of the entire RRegrs methodology in a single line. All details about functions’ main parameters are given in the R package documentation. All these parameters have default values. The default values imply a default location for the output files, execution of all modelling steps (removal of NA, and near zero variance features, and of correlated features), normalization of the data set, ten splits, ten Y-randomization steps, and running of all ten regression methods. The user can alter any step or parameter of the RRegrs methodology.

The following examples show simple calls of the RRegrs() function using a specific dataset file entitled ”MyDataSet.csv” that it should be provided by the user:



The output variable RRegrsResults is a complex object which contains the object of the fitted models and the main statistics for each regression model. Details about each function are presented into the tutorial of the RRegrs package.

The following example could be used to test the RRegrs package using a dataset file from RRegrs GitHub URL:



### Availability and requirements

- Project name: RRegrs
- Project home page: RRegrs
- Operating system(s): Platform-independent
- Programming language: R programming language
- Other requirements: R 3.1.0 or higher
- License: NewBSD or MIT
- Any restrictions to use by non-academics: none other than those defined by the license

## Abbreviations

RRegrs: R regressions (package)
QSAR: quantitative structure-activity relationship
R^2^: R-squared
RMSE: root-mean-square error
AIC: akaike information criterion
LM: linear multi-regression
GLM: generalized linear model with stepwise feature selection
PLS: partial least squares regression
LASSO: Lasso regression
ENET: elastic net regression
SVRM: support vector machine using radial functions
NN: neural networks regression
RF: random forest
RF-RFE: random forest recursive feature elimination
SVM-RFE: support vector machines recursive feature elimination
CSV: comma-separated values file format
PDF: portable document format of files
PNG: portable network graphics file format
NP: nanoparticle
